# Multiplexed monitoring of a novel autoantibody diagnostic signature of colorectal cancer using HaloTag technology-based electrochemical immunosensing platform

**DOI:** 10.7150/thno.42507

**Published:** 2020-02-10

**Authors:** María Garranzo-Asensio, Ana Guzmán-Aránguez, Eloy Povedano, Víctor Ruiz-Valdepeñas Montiel, Carmen Poves, María Jesús Fernandez-Aceñero, Ana Montero-Calle, Guillermo Solís-Fernández, Servando Fernandez-Diez, Jordi Camps, Meritxell Arenas, Elisabeth Rodríguez-Tomàs, Jorge Joven, Maricruz Sanchez-Martinez, Nuria Rodriguez, Gemma Dominguez, Paloma Yáñez-Sedeño, José Manuel Pingarrón, Susana Campuzano, Rodrigo Barderas

**Affiliations:** 1 Departamento de Bioquímica y Biología Molecular, Facultad de Óptica y Optometría, Universidad Complutense de Madrid, 28037 Madrid, Spain.; 2 UFIEC, Chronic Disease Programme, Instituto de Salud Carlos III, Majadahonda 28220, Madrid, Spain.; 3 Departamento de Química Analítica, Facultad de Ciencias Químicas, Universidad Complutense de Madrid, 28040 Madrid, Spain; 4 Gastroenterology Unit, Hospital Universitario Clínico San Carlos, E-28040, Madrid, Spain; 5 Pathology Department, University Hospital Gregorio Marañon, E-28007 Madrid, Spain; 6 Unitat de Recerca Biomèdica, Hospital Universitari Sant Joan, Institut d´Investigació Sanitària Pere Virgili, Universitat Rovira i Virgili, Reus (Spain).; 7 Department of Radiation Oncology, Hospital Universitari Sant Joan, Institut d´Investigació Sanitària Pere Virgili, Universitat Rovira i Virgili, Reus (Spain).; 8 Medical Oncology Department, Hospital Universitario La Paz, E-28046, Madrid, Spain.; 9 Departamento de Medicina, Facultad de Medicina, Instituto de Investigaciones Biomédicas "Alberto Sols", CSIC-UAM, E-28029, Madrid, Spain.

**Keywords:** colorectal cancer, biosensor, early cancer detection, electrochemical bioplatforms, halotag fusion proteins, liquid biopsy

## Abstract

**Background and Purpose**: The humoral immune response in cancer patients can be used for early detection of the disease. Autoantibodies raised against tumor-associated antigens (TAAs) are promising clinical biomarkers for reliable cancer diagnosis, prognosis, and therapy monitoring. In this study, an electrochemical disposable multiplexed immunosensing platform able to integrate difficult- and easy-to-express colorectal cancer (CRC) TAAs is reported for the sensitive determination of eight CRC-specific autoantibodies.

**Methods**: The electrochemical immunosensing approach involves the use of magnetic microcarriers (MBs) as solid supports modified with covalently immobilized HaloTag fusion proteins for the selective capture of specific autoantibodies. After magnetic capture of the modified MBs onto screen-printed carbon working electrodes, the amperometric responses measured using the hydroquinone (HQ)/H_2_O_2_ system were related to the levels of autoantibodies in plasma.

**Results**: The biosensing platform was applied to the analysis of autoantibodies against 8 TAAs described for the first time in this work in plasma samples from healthy asymptomatic individuals (n=3), and patients with high-risk of developing CRC (n=3), and from patients already diagnosed with colorectal (n=3), lung (n=2) or breast (n=2) cancer. The developed bioplatform demonstrated an improved discrimination between CRC patients and controls (asymptomatic healthy individuals and breast and lung cancer patients) compared to an ELISA-like luminescence test.

**Conclusions**: The proposed methodology uses a just-in-time produced protein in a simpler protocol, with low sample volume, and involves cost-effective instrumentation, which could be used in a high-throughput manner for reliable population screening to facilitate the detection of early CRC patients at affordable cost.

## Introduction

Colorectal cancer (CRC) is the second deadliest cancer worldwide because of its late diagnosis. The 5-year survival rate associated to late diagnosis drops to 6-10% 1. The study of the humoral immune response has been demonstrated useful to identify tumor-associated antigens (TAAs) in CRC with diagnostic ability. Autoantibodies against TAAs appear up to three years before clinical symptoms because of the immune system amplification, making them interesting targets for early cancer detection 2-6. Early CRC diagnosis by population screening would increase overall patient well-being, as well as have an important impact on overall Health Systems since 90% of early diagnosed patients are successfully cured.

By protein and phage microarrays, among other approaches, numerous CRC TAAs whose autoantibodies have potential diagnostic ability have been described 4, 7-12. However, the detection of autoantibodies to some of these TAAs had to be discarded because they were difficult-to-express and/or purify proteins because of degradation or aggregation during purification or storage. These problems make it very difficult and time-costly (if possible) to include them into multiplexed diagnostic panels. Thus, the development of multiplexed biosensing strategies coupled to *in vitro* expression systems for autoantibody detection able to overcome such problems would be of great interest. On the one hand, mammalian cellular extracts would be ideal since it would allow the proteins to be expressed in a short period of time with correct folding and no degradation to ensure its functionality. On the other hand, current electrochemical biosensing platforms, entailing straightforward processes and capable of accurately detecting specific targets in scarcely treated biofluids represent an interesting alternative to conventional immunoassays. Their versatility, amenability to detect numerous molecular targets simultaneously with high levels of sensitivity and specificity, capacity for automation, affordability, portability and minuscule amount of sample requirement make them ideal candidates to be adapted in clinical routine for point-of-care (POC) diagnosis of cancer and other prevalent diseases 13-28.

This work reports the construction of an electrochemical immunosensing platform using *in vitro* transcription/translation expressed HaloTag fusion proteins self-assembled onto commercial magnetic microparticles (MBs). HaloTag is an engineered dehalogenase developed to covalently bind to halogenated alkanes 29, 30, which in addition can improve the solubility of fusion proteins providing higher yields, purity and overall recovery of the expressed proteins in comparison to other tags as FLAG, 3×FLAG or His(6)Tag 30. Amperometric detection at screen-printed carbon electrodes (SPCEs) was performed to evaluate the diagnostic potential of a novel autoantibody panel composed of eight TAAs (GTF2B, MAPKAPK3, PIM1, PKN1, SRC, STK4, SULF1, and p53) previously validated or not validated for CRC diagnosis because of their difficulty to be expressed or purified 7, 9. It is worth to remark that in a previous work, we reported the use of MBs coupled to *in situ* expressed HaloTag fusion proteins and electrochemical detection at SPCEs for the accurate determination of blood autoantibodies. However, this technology was applied only to the determination of antibodies against a single TAA (p53) 13. This work showed that the HaloTag fusion protein-based electrochemical immunosensing was less prone to false results than ELISA involving recombinant TAAs produced in bacterial hosts. This fact was attributed to the differences in concentration, type, and immobilization of bioreceptors (p53 expressed in bacteria vs HaloTag-p53 expressed in a mammalian milieu) which certainly affected the autoantibody capture efficiency, and to the remarkably higher sensitivity (440 times) and the larger sera dilution factor required by the electrochemical method compared to the ELISA test. Interestingly, the analysis of the difficult-to-express TAAs PKN1, SRC, and SULF1 could not be carried out by ELISA and/or Luminex methods. Moreover, PIM1 forms dimers that can block antibody recognition sites and, therefore, if aggregation occurs becomes useless for patient diagnosis, as in Luminex-based approaches 7. In addition, MAPKAPK3, STK4, and p53 were included as controls to evaluate whether these TAAs maintain their previously described diagnostic potential 7, 9. *In vitro* expressed TAAs coupled to HaloTag were immobilized *in situ* into MBs to avoid protein degradation. Then, modified MBs were incubated with plasma samples from controls, CRC patients, or colorectal individuals carrying premalignant lesions to assess the diagnostic ability of the autoantibodies. The developed biosensing multiplexed platform can be envisioned as a point-of-care (POC) device capable of detecting these autoantibodies in plasma.

## Results

### Colorectal cancer tumor-associated antigens selection

Previous studies related to the analysis of the humoral immune response in CRC identified 91 possible TAAs by using protein microarrays. Among them, difficult-to-express and difficult-to-purify TAAs or autoantigens suffering from extensive degradation with high diagnostic potential were discarded for analysis and/or validation. Therefore, only a panel consisting of 6 TAAs was considered for early CRC detection 4, 7, 10, 11.

In this work, we have developed an electrochemical immunosensing strategy based on HaloTag technology able to include previously discarded proteins, due to their challenging production and/or purification, into diagnostic panels with the ultimate goal of constructing a POC device able to discriminate CRC patients and individuals carrying premalignant lesions from controls. For such a purpose, eight CRC TAAs were selected for the study: GTF2B, MAPKAPK3, PIM1, PKN1, SRC, STK4, SULF1, and p53, where PIM1, PKN1, SRC, and SULF1 (and p53) are difficult-to-express and/or purify proteins and GTF2B, MAPKAPK3, and STK4 are easy-to-express proteins used as controls to ascertain whether this approach maintains their previously described CRC diagnostic ability 7, 9.

**Figure 1:**
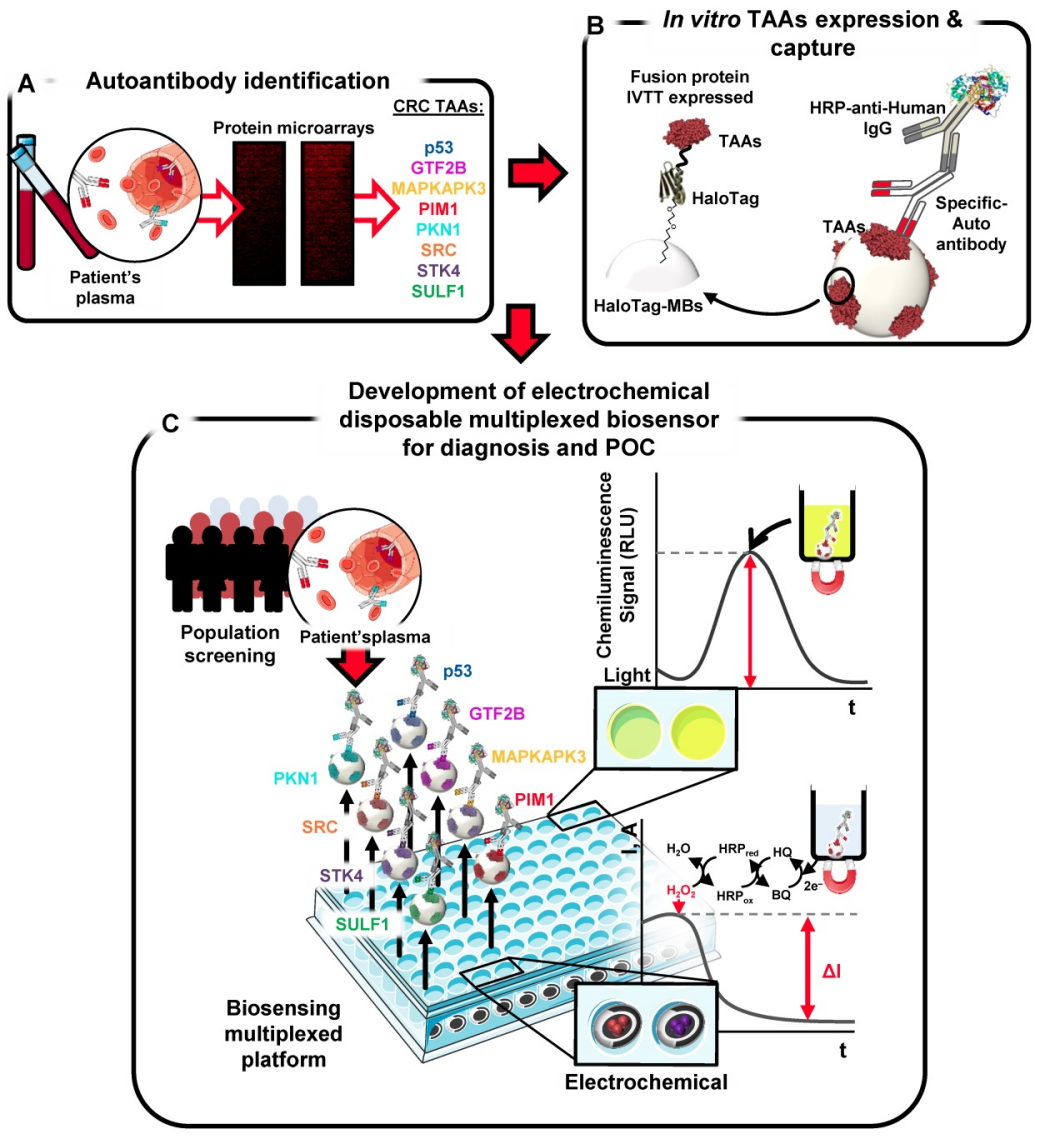
** Schematic design of the strategy.** Autoantibody targets were identified using protein microarrays incubated with plasma samples from CRC patients. Targets were then *in vitro* expressed as HaloTag fusion proteins for their subsequent immobilization onto MBs for evaluation of their diagnostic potential and development of a multiplexed electrochemical immunosensing platform for CRC early diagnosis.

CRC TAAs encoding cDNAs were cloned as HaloTag-fusion proteins into vectors optimized for *in vitro* protein expression for subsequent covalent immobilization of the proteins onto MBs for direct evaluation of autoantibody levels in plasma of patients and controls (Table 1 and Table S1) to avoid any degradation or precipitation during purification and storage, compulsory steps for other techniques (i.e. ELISA, Luminex…) 13. Once verified the correct protein expression and established the diagnostic signature by luminescence 31, a multiplexed electrochemical immunoassay capable of detecting autoantibody presence as a POC device for CRC detection was developed (Figure 1).

### Cloning and *in vitro* protein expression

To evaluate whether the proposed system of *in vitro* protein expression and immobilization of the autoantibody targets onto MBs could successfully render all TAA fusion proteins, we evaluated their correct *in vitro* expression. Figure S1A shows as immunostaining of the expression product exhibited a correct expression. Next, to verify that the HaloTag fusion proteins were functional for their covalent binding to functionalized chloroalkane MBs, and thus, could be used for selective capture of autoantibodies against them, we immobilized them onto MBs through the HaloTag and incubated the resulting HaloTag fusion protein-MBs with, alternatively, a specific antibody against the tag or against the indicated TAAs. A specific luminescence signal was obtained for all proteins using the anti-HaloTag mAb (Figure S1B) or for the indicated TAAs using their specific antibodies (Figure S1C). These results confirmed the successful immobilization of functional HaloTag fusion proteins on the MBs and the great potential of the protocols applied to any protein without particular optimization.

### Evaluation of autoantibody seroreactivity levels by luminescence beads immunoassay

**Figure 2:**
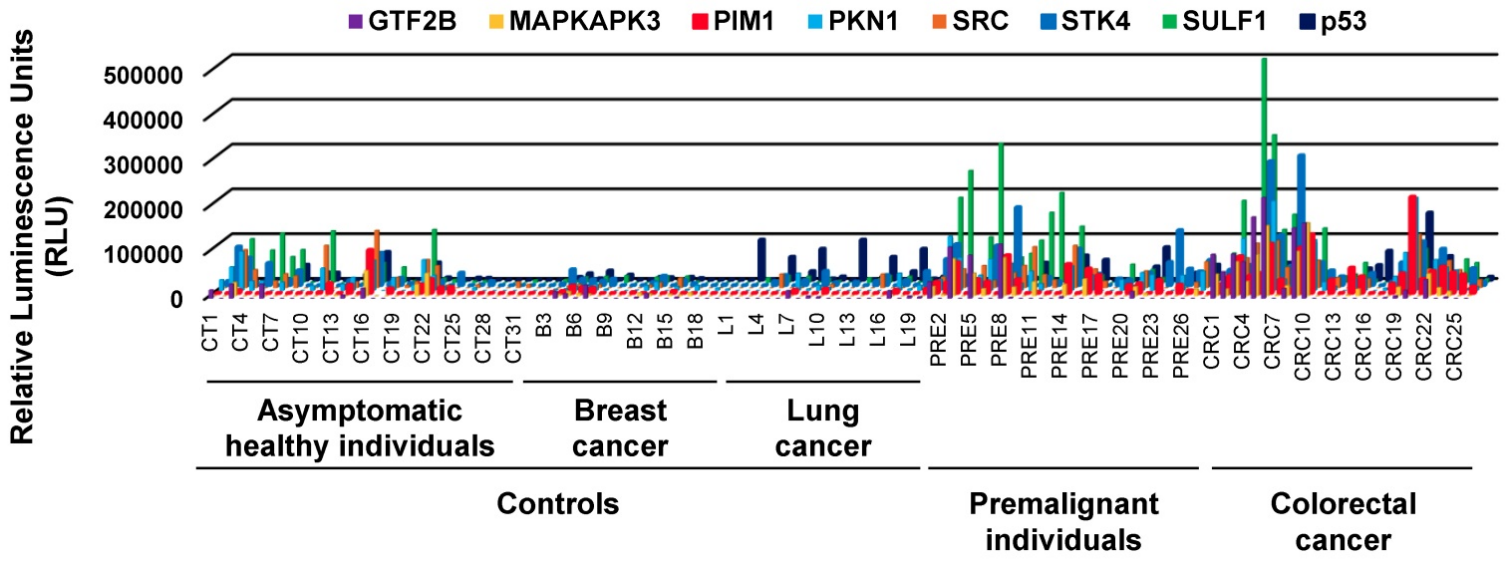
** Autoantibody measurement by luminescence of the plasma samples.** Evaluation of autoantibody presence showed that the luminescence signal obtained for the premalignant and CRC groups was considerably higher than the signal obtained for the control groups.

Once established the optimal working conditions, we evaluated the optimal plasma dilution to be used by luminescence beads immunoassay to compare the results with those previously reported for GTF2B, MAPKAPK3, and STK4 7, 9. Figure S1D shows as better discrimination between the tumor and control group for the determination of autoantibodies a 1:300 dilution. Next, we proceeded to evaluate the seroreactivity levels of 125 individuals -31 asymptomatic healthy individuals, 20 lung cancer patients, 20 breast cancer patients, 28 premalignant individuals, and 26 CRC patients- to determine whether autoantibody presence could discriminate between groups, and to assess whether the developed methodology was successful to be used with difficult-to-express and easy-to-express CRC TAAs proteins for diagnostic purposes (Figure 2).

**Figure 3:**
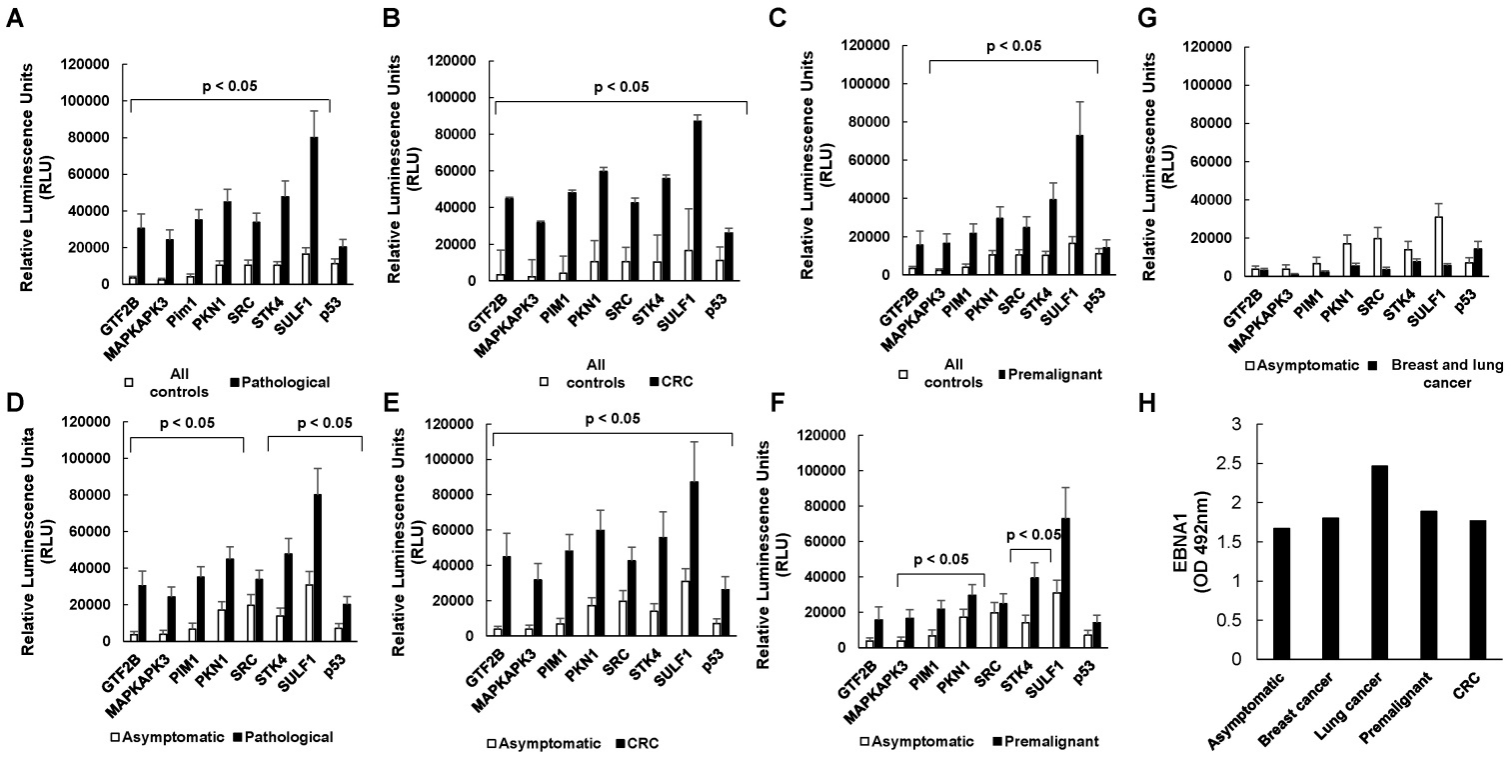
** Statistical analysis of autoantibody levels according to patients' groups.** Autoantibodies against the eight targets could discriminate between the control and pathological group (**A**) and between controls and CRC patients (**B**). However, GTF2B and p53 could not discriminate between the control and premalignant subjects (**C**). All the autoantibodies but SRC could discriminate between the asymptomatic subjects and the pathological group (**D**). Moreover, the 8 autoantibodies could discriminate between the asymptomatic individuals and CRC patients (**E**). However, only MAPKAPK3, PIM1, PKN1, and STK4 could discriminate between asymptomatic and premalignant subjects (**F**). Furthermore, just p53 could differentiate between the asymptomatic individuals and breast and lung cancer patients (**G**). EBNA-I seroreactivity was analyzed as a test for the specificity of the assay, showing similar levels in all groups (H). Since >95% of the human population has been infected with the Epstein Barr virus, antibodies specific against EBNA1 would serve as specific control of the seroreactivity among all the analyzed groups. All controls: asymptomatic individuals, and breast and lung cancer patients.

Overall, we found that the evaluated autoantigens target of autoantibodies -except p53- could discriminate with statistical significance (p<0.05) among the pathological individuals (CRC patients and premalignant individuals) and all control groups, as well as all controls *vs* CRC patients (Figure 3A, B). However, when comparing the control and premalignant patients, GTF2B and p53 failed to distinguish between groups with statistical significance even though the premalignant group showed higher autoantibody levels (Figure 3C). When comparing the autoantibody levels in asymptomatic individuals and the pathological group, all the autoantibody targets except SRC could significantly discriminate between groups, while all autoantibody targets could discriminate between them with statistical significance when comparing the asymptomatic individuals with the CRC patients (Figure 3D, E). However, GTF2B, SRC, SULF1 and p53 could not discriminate between the asymptomatic and premalignant subjects with statistical significance although the premalignant group showed higher autoantibody levels (Figure 3F). To evaluate whether autoantibodies were CRC specific, we compared levels between the asymptomatic subjects and breast and lung cancer individuals. Autoantibodies against p53 could discriminate with statistical significance (p<0.05) between both groups but none of the CRC TAAs (Figure 3G).

EBNA1 seroreactivity was analyzed as control of the specific CRC seroreactivity. A no clear pattern of seroreactivity to EBNA1 was observed among all groups with non-significant p-values. These results clearly confirmed that the seroreactivity observed for the indicated CRC-specific autoantibodies was characteristic of the analyzed groups (Figure 3H).

### Evaluation of the diagnostic potential of selected autoantibodies

**Figure 4:**
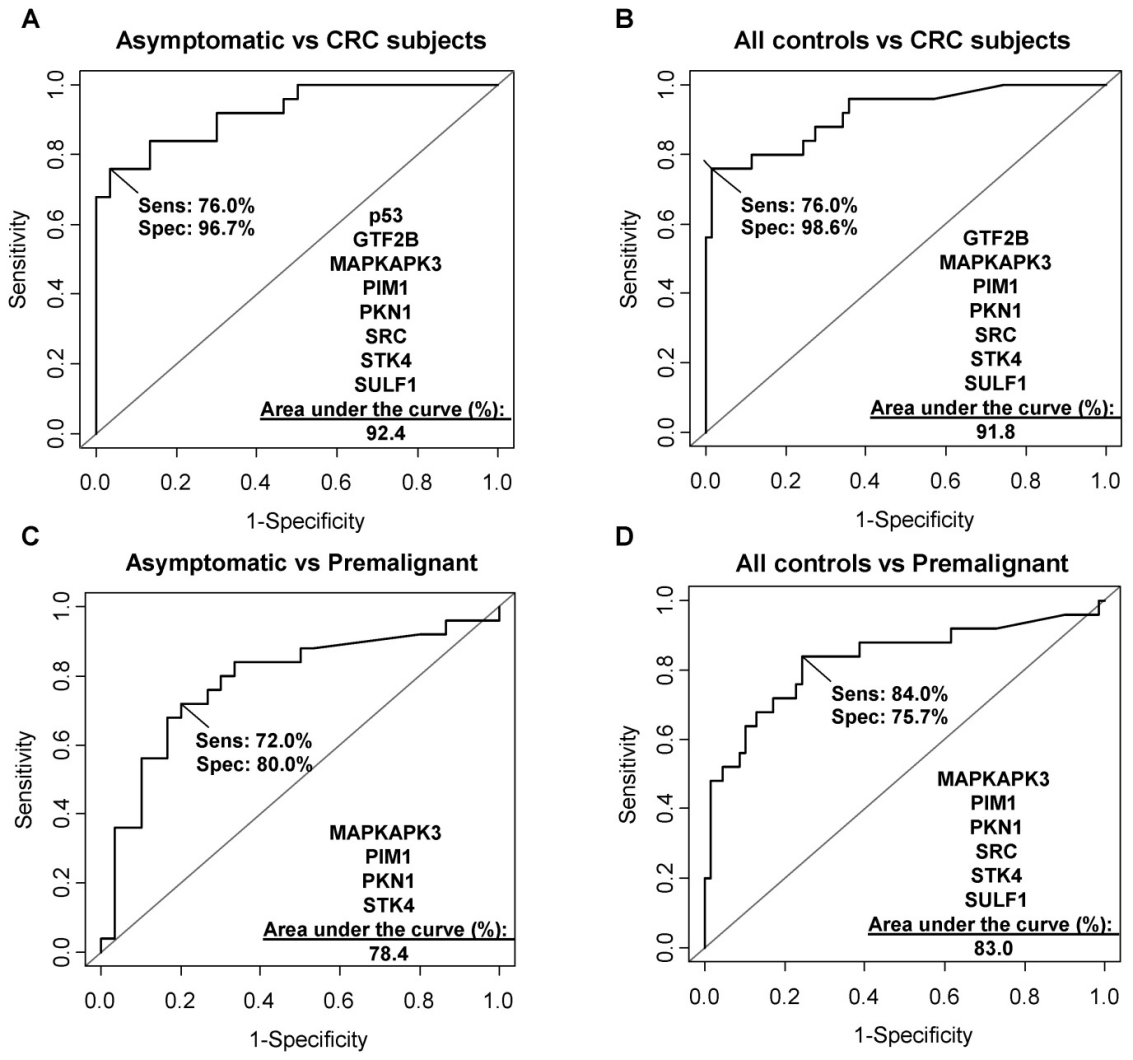
** Diagnostic potential of the simultaneous detection of the TAAs.** The diagnostic potential was analyzed by ROC curves comparing the asymptomatic *vs* CRC group (**A**), all controls *vs* CRC subjects (**B**), asymptomatic individuals *vs* premalignant subjects (**C**), and all controls *vs* the premalignant group (**D**). All controls: asymptomatic individuals, and breast and lung cancer patients.

Next, ROC curves were obtained to search for the optimal TAA combination to discriminate between patients and controls (Table S2). The optimal AUC values were achieved with those autoantibodies that could discriminate patients with statistical significance, with a value of 92.4% (sensitivity 76.0%, specificity 96.7%) when comparing the asymptomatic group vs the CRC patients, and 91.8% (sensitivity 76.0% and specificity 98.6%) when factoring in the breast and lung cancer subjects with the asymptomatic group against the CRC subjects (Figure 4A, B).

To evaluate whether the proposed markers could also detect premalignant subjects, ROC curves comparing the asymptomatic group with the premalignant subjects as well as the control group (with the breast and lung cancer subjects) with the premalignant subjects were constructed. AUC values of 78.4% (sensitivity 72%, specificity 80%) and 83.0% (sensitivity 84%, specificity 75.7%) were found, respectively (Figure 4C, D). These AUC values, although lower than those obtained for discriminating the CRC subjects, also indicated that the markers are useful for the early detection of premalignant individuals.

These data were compared with those previously reported for GTF2B, STK4, MAPKAPK3, and p53 to ascertain whether this approach keeps their previously reported CRC diagnostic values (Table S2, and Table S3). Our data showed that the here proposed approach is suitable for difficult-to-express and difficult-to-purify TAAs as well as for those easy-to-produce. In this sense, GTF2B, STK4, MAPKAPK3 and p53 showed a similar or even higher diagnostic ability using the here described approach than that previously reported (Table S3), which may be attributed to the lower false positives and the high sensitivity demonstrated by the HaloTag technology compared to ELISA 4, 7, 9, 11, 13.

### Development of an electrochemical multiplexed immunosensing platform as a tool for early and affordable diagnostic test

**Figure 5:**
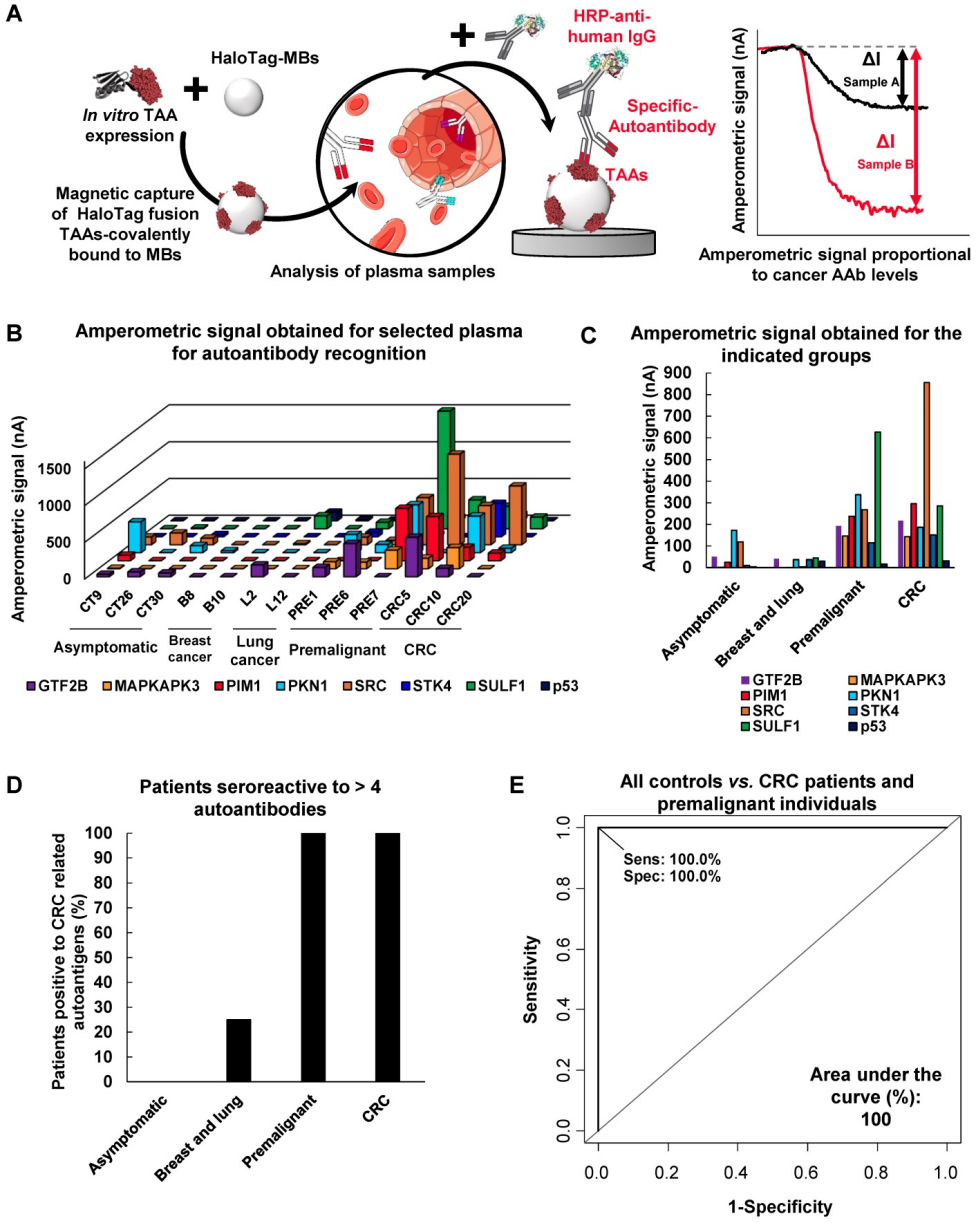
** Autoantibody measurement in plasma samples by the multiplexed electrochemical immunosensing platform.** (**A**) Schematic design of the proposed strategy. (**B, C**) Amperometric responses obtained for the TAAs were larger for the premalignant and CRC subjects in comparison to the asymptomatic, where they were almost undetectable. (**D**) Percentage of patients positive to CRC related autoantibodies detected by the biosensor. (**E**) ROC curve of autoantibody detection against all targets using the electrochemical immunosensing platform. Premalignant, premalignant colorectal individuals.

The CRC autoantibody targets were also tested by means of a multiplexing electrochemical immunosensing platform as a proof-of-concept of a POC device that could be used in a clinical setting for CRC detection (Figure 5A).

Under the optimized conditions described in the Experimental Section, the ability of the developed platform to detect the presence of autoantibodies against GTF2B, MAPKAPK3, PIM1, PKN1, SRC, STK4, SULF1, and p53 in human plasma was tested. Three asymptomatic plasma samples, samples from three premalignant individuals, three CRC patients and four breast and lung cancer patients were analysed (Figure 5B, C). As it was previously observed by luminescence, most CRC patients were reactive to most if not all TAAs, while premalignant individuals were reactive to >4 TAAs apart from p53.

We analyzed premalignant individuals, CRC patients and control groups according to this differential specific seroreactivity as cut-off. Remarkably, none of the asymptomatic patients (with most values close to background) and breast cancer patients showed seroreactivity to four or more different CRC autoantigens and were clearly discriminated from CRC patients. However, one lung cancer patient also presented seroreactivity to 4 out of 8 different CRC autoantigens. In addition, all premalignant and CRC individuals possessed autoantibodies for more than four CRC autoantigens and showed a clear signal above background, indicating the usefulness of the methodology as a possible clinical tool for the detection of CRC (Figure 5D). Furthermore, ROC curve analysis showed that the multiplexed simultaneous detection of autoantibodies against the eight CRC TAAs has a powerful diagnostic ability for CRC and colorectal premalignant individuals, with an AUC of 100% (Figure 5E).

Collectively, these results show that the developed methodology overcomes the drawbacks regarding purification, degradation and immobilization of difficult-to-express CRC TAAs so that they do not have to be discarded with the implicated loss of diagnostic ability. Moreover, the use of these TAAs allowed us to find a novel diagnostic signature with high potential for the detection of CRC through a POC device.

## Discussion

The humoral immune response has been proven useful for early cancer diagnosis 2, 3, 5, 32-34. Through different methodologies, such as ELISA, SEREX, SERPA, or other proteomic techniques, multiple potential tumor associated targets of the immune system have been identified 8, 11, 12, 35-37. However, these methodologies are not ideal for validation or to be used in a clinical setting, since they require either large volumes of biological samples or are expensive and time-consuming when they have to detect multiple targets simultaneously, or require a high specialized setting. In this work, we have optimized a novel methodology based on the *in vitro* expression of CRC-specific autoantibody targets coupled to HaloTag that allows their immobilization onto MBs and the construction of an electrochemical immunosensing platform with multiplexing capabilities ideal as a POC device for the analysis in serum/plasma.

*In vitro* protein expression using mammalian cellular extracts offers numerous advantages versus the most common bacteria and yeast expression systems. As it has been shown in previous studies, some TAAs in CRC exhibited expression and purification drawbacks which made it hard to develop with them a methodology for CRC diagnosis 4, 7, 9-11. The expression of the autoantibody targets coupled to the HaloTag using a previously developed system for p53 made it possible to obtain the difficult-to-express proteins previously discarded with their correct folding and no degradation, and in less time than with conventional techniques. Moreover, through the HaloTag, these functionalized proteins were covalently immobilized *in situ* to carry out the assay, so that no storage or dimerization problems occurred and autoantibody detection was not hindered by their degradation or oligomerization. It is important to note also that this protocol for *in situ* production and selective capture of the HaloTag fusion proteins onto the MBs is much simpler and shorter (just one overnight step) than the conventional procedure to get purified TAAs produced in bacteria, insect, or mammalian cells, which usually requires weeks or months, and sometimes makes it impossible to produce difficult-to-express or -purify proteins and are more prone to give false positive results due to the presence of bacterial contaminants.

Autoantibody detection in plasma samples is an interesting alternative to conventional diagnostic techniques for CRC -such as colonoscopy- since it is a non-invasive (or low- invasive since blood collection is required) approach that can detect the disease at either premalignant (low- and high-grade adenomas) and CRC at early stages when its treatment get more than 90% of curation only with tumor resection. Up to date, autoantibody levels have been described to be useful for the diagnosis of multiple different diseases 35, 38-40, apart from other cancer malignancies 41, 42, and thus, this methodology could also be applied for different diseases.

In this work, we found that the simultaneous detection of autoantibodies against GTF2B, MAPKAPK3, p53, PIM1, PKN1, SRC, STK4, and SULF1 could discriminate between control individuals and CRC patients and premalignant subjects with an AUC well above than that for CEA (the established clinical marker recommended for monitoring recurrent CRC) 90% *vs* 80%, and especially for the comparison between control individuals and premalignant patients 91% *vs* 57%. Moreover, this autoantibody panel was specific of CRC, since it could not discriminate individuals with breast and lung cancer patients, indicating their great potential in a clinical setting.

Electrochemical biosensors are attractive potential diagnostic tools, since they offer numerous advantages in comparison to other techniques such as ELISA or Luminex 13, 43, 44. They have a great multiplexing capability, with ability to detect different biomarkers in nature in a single measurement and directly in complex biological matrices 43. This characteristic makes this type of biosensors ideal for CRC diagnosis since it would allow for the simultaneous autoantibody detection against the eight TAAs using a minimal plasma sample volume. The multiple detection of autoantibodies is needed for a more specific and sensitive diagnosis of the disease, and to discriminate CRC patients from other cancer patients. As shown in this work, the ROC curves of the biosensing approach demonstrated that this methodology could be successfully used in clinics to detect CRC patients. In addition, the developed methodology meets better the requirements of hospital routine and POC testing since it greatly reduces assay time and production costs. In comparison to other techniques, the simultaneous detection of the autoantibody presence takes up to 3.5 hours after protein immobilization on chloroalkane MBs. This assay time is much shorter than that required by using ELISA or Luminex for testing the same number of TAAs (about 6 hours after protein immobilization without considering the overnight immobilization of the TAAs by ELISA or the 3 days required after protein immobilization by Luminex prior to seroreactive analysis). Importantly, the multiplexed electrochemical biosensing for 8 TAAs detection is about 3 euros per patient that would be 10-20 times less expensive than other conventional immunoassays. In addition, the TAAs used for selective capture of the autoantibodies are expressed just-in-time using a cell-free expression system, allowing for the first time the determination of autoantibodies against TAAs difficult to express and/or purify. Furthermore, this methodology avoids the expensive and time demanding protocols required to express and purify cancer recombinant proteins in bacterial, insect, or mammalian cells and the concerns regarding the stability and integrity of the protein during storage, as well as the presence of potentially immunogenic bacterial products that can be a source of false positives in ELISA or Luminex assays.

Besides the use of HaloTag technology, the proposed electrochemical immunosensing platform possesses unique features, in comparison with ELISA, derived from the use of MBs as solid supports to perform the bioassay and the electrochemical transduction at disposable electrodes. MBs have demonstrated to be a powerful and versatile tool to improve the sensitivity of bioassays, minimize matrix effect, reduce largely the assay time and make analytical procedures more compatible with higher sample throughput and automation 45-49. Screen-printed electrodes (SPEs), can be massively and inexpensively produced from a variety of materials, in different geometries and in miniaturized and multiplexed formats and allow working with small sample volumes 47, 50. Moreover, the developed technology is amenable to automate and implement using cost-effective and low-power requirement instrumentation, which make it applicable in both diagnostic and outpatient routines and even in limited-resource settings 23. All these interesting features and the ability to discriminate between patient groups, make the developed bioplatform a promising tool compared both to ELISA and the luminescence approach to be readily implemented in low-cost, simple use, short time analysis and in high-throughput and multiplexed devices for early diagnostics, patient follow-up, and monitoring of cancer patients through reliable autoantibodies signatures determination. However, the main issue the developed electrochemical methodology would have to face might be precisely to convince of the potential and these competitive advantages to the users of ELISA technology, very accustomed with using this methodology long adopted in centralized laboratories for this type of determinations.

In conclusion, in this work we have designed and optimized a novel and versatile electrochemical biosensing strategy compatible with POC demands for the detection of multiple TAAs, even those difficult-to-be-expressed, based on the use of HaloTag fusions proteins obtained by *in vitro* expression systems maintaining or increasing their diagnostic ability. The autoantibodies against the described TAAs could discriminate CRC and premalignant subjects from control individuals with great specificity and sensitivity. The capability of the developed technology was demonstrated by performing 104 and 1,000 determinations using electrochemical and chemiluminescence detection, respectively. The results obtained using both detection techniques were comparable and showed that while no seroreactivity was observed against any of the tumor antigens selected in asymptomatic patients, 100% of patients with premalignant lesions or CRC exhibit seroreactivity to at least 4 out of the 8 selected antigens. These results confirm the possibility of reliably and minimally invasively diagnose CRC by analyzing molecular signatures comprising these eight autoantibodies. Although further improvements could be made by including other CRC TAAs to increase the diagnostic ability of the developed electrochemical technology, and further validations using larger independent patient's cohorts, the capabilities and advantages demonstrated so far reveal its potential to integrate difficult- and easy-to-express TAAs to contribute to early and reliable CRC diagnosis, and thus greatly increasing patient income and benefiting the cost associated to CRC treatment by Health Systems.

## Materials and methods

### Plasma samples

The Institutional Ethical Review Boards of the Instituto de Salud Carlos III, Hospital Clínico San Carlos, and La Paz Hospital approved this study on biomarker discovery and validation (CEI PI 45). Plasma samples (Table 1 and Table S1) were used accomplishing all the ethical issues and relevant guidelines and regulations.

Plasma samples (Table 1 and Table S1) were obtained from the biobanks of the Hospital Clínico San Carlos and Hospital La Paz after approval of the Ethical Review Boards of these institutions. Breast and lung cancer samples provided by Hospital Universitari de Sant Joan (Tarragona, Spain) were used accomplishing all the ethical issues and relevant guidelines and regulations. All subjects in the study gave their written informed consent to participate and all experiments were performed in accordance with relevant guidelines and regulations.

For the analysis of the CRC diagnostic ability of autoantibodies, a panel of 125 plasma samples from colorectal cancer patients and premalignant colon subjects (low- and high-grade adenomas), and control individuals (asymptomatic healthy and negative colonoscopy individuals, Fecal Occult Blood Test -FOBT- positive and colonoscopy negative individuals, and breast and lung cancer patients) was used (Table 1, and Table S1). Plasma samples were collected using a standardized sample collection protocol and stored at -80°C until use 4, 9-11.

### Gateway plasmid construction, gene cloning, DNA preparation and protein expression

Sequence-verified, full-length cDNA plasmids containing selected targets for validation in flexible pDONR221 or pENTR223 vector system were obtained from the publicly available DNASU Plasmid Repository (https://dnasu.org/DNASU/) 51. The ORFs were transferred by LR clonase reactions (Invitrogen, Carlsbad, CA), alternatively, to a pANT7_cHalo or pJFT7_nHalo vector for *in vitro* protein expression to get the autoantigens expressed as fusion proteins to HaloTag in the C-terminal or in the N-terminal, respectively 13, 52. All donor and expression plasmids were sequence verified prior to a subsequent use.

To obtain high-quality supercoiled DNA, plasmids were transformed into TOP10 E. coli cells and grown in 250 mL Luria Bertani (LB) supplemented with the appropriate antibiotic (100 μg/mL Ampicilin and 40 μg/mL kanamycin). Plasmid DNA was purified using the NucleoBond Xtra Midi kit (Macherey-Nagel Inc., Bethlehem, PA). Proteins were cell-free expressed *in vitro* using HeLa cell lysate from the 1-Step Human Coupled IVT Kit (Thermo Fisher Scientific, Waltham, MA) per manufacturer's recommendations to carry out the ELISA studies.

### SDS-PAGE and western blot analysis

SDS-PAGE and western blot analysis to assess protein quality was performed as previously reported 53. Briefly, 0.67 µL of the *in vitro* protein extracts were run in 10% SDS-PAGE and transferred to nitrocellulose membranes (Hybond-C extra). After blocking, membranes were incubated overnight at 4 °C with an anti-HaloTag monoclonal antibody. Immunodetection on the membranes was achieved using HRP-conjugated goat anti-mouse IgG antibody (Table S4). Chemiluminescence signal was developed with ECL Western Blotting Substrate (Thermo Scientific) and detected on an Amersham Imager 680 (GE Healthcare).

### EBNA-1 ELISA

Colorimetric ELISA for EBNA-I antibody determination for the evaluation of the CRC autoantibody response specificity was accomplished by coating 0.05 µg of EBNA-I protein kindly provided by Protein Alternatives, S.L. per well in 50 µL of phosphate-buffer saline solution (PBS) in 96-well Maxisorp plates (Nunc) overnight at 4 ºC. Plates were then blocked using a 3% (w/v) skimmed-milk solution in PBS supplemented with 0.1% Tween (PBST) for 1 h at 37 ºC and then incubated 1 h at 37ºC with 50 µL of the 1:300 diluted plasma samples. After extensive washing with PBST, plates were incubated for 1 h at 37 ºC with 50 µL of an HRP-labeled secondary antibody. Colorimetric signal was developed as previously described 54, 55.

### Autoantibody analysis by luminescence beads immunoassay

Protein coupling to Magne HaloTag beads (MBs, Promega) was performed overnight at 4 ºC and 1000 rpm according to the manufacturer instructions using 0.67 µL of IVT expression reaction and 0.5 µL of HaloTag MBs per measurement. For covalent binding, a mixture of the required amount of protein and MBs was made taking into account the number of replicates and measurements to be performed 13, 35, 56. After extensive washing with PBS, Tween 0.1%, Triton X-100 0.05% using a magnet and removal of non-covalently bound proteins with 0.1 M glycine, pH 2.7, HaloTag fusion proteins immobilized onto MBs were blocked with Superblock (Pierce) for 1 h. To verify covalent protein immobilization, the HaloTag fusion proteins were detected with either anti-HaloTag (Promega), or specific antibodies against the TAAs followed by 1 h incubation with the corresponding diluted HRP conjugated secondary antibody (Table S4). Alternatively, they were incubated overnight with pooled or individual plasma samples at indicated dilutions at 1000 rpm and 4 ºC. After washing with PBS, Tween 0.1%, Triton X-100 0.05% as above, HRP-conjugated anti-human IgG antibody (Dako) 1:10000 diluted in PBS supplemented with Tween 20 0.1% (v/v) and 3% (w/v) BSA was incubated with the MBs. The MBs were collected and placed on black Maxisorp 96-well plates (Nunc). Signal was developed using 50 μL of SuperSignal ELISA Femto Maximum Sensitivity Substrate (Pierce, Rockford, IL) for the detection of luminescence on The Spark multimode microplate reader (Tecan Trading AG, Switzerland). Alternatively, plates were also recorded on an Amersham Imager 680 (GE Healthcare).

### Autoantibody analysis by electrochemical beads immunoassay

The protocol was previously reported and optimized for the determination of p53 autoantibodies 13. The same protocol was followed for the determination of CRC-specific autoantibodies against GTF2B, MAPKAPK3, PIM1, PKN1, SRC, STK4, SULF1, and p53. To verify the correct protein immobilization, the HaloTag fusion proteins were detected with anti-HaloTag mAb (Promega), followed by 1 h incubation with 1:3000 diluted HRP-conjugated anti-mouse IgG (Sigma Aldrich). The amperometric measurements were performed in the presence of the hydroquinone (HQ)/H_2_O_2_ system 57 at disposable screen-printed carbon electrodes (SPCEs) upon magnetic capture of the MBs bearing the immunocomplexes on the working electrode 13. The biorecognition event is monitored by the variation in the cathodic current generated by the enzymatic (HRP) reduction of H_2_O_2_ mediated by HQ and measured at -0.20 V (vs a Ag pseudoreference electrode) 57.

The amperometric measurements were carried out with a single SPCE for each measurement (that do not take more than 3 min) and discarded afterwards. However, no significant change in the working protocol would be required to measure simultaneous signals at arrays of 8 electrodes or even at electrochemical plates of 96 electrodes.

### Statistical analysis

All statistical analyses were done with Microsoft Office Excel and the R program. For the analysis of the results obtained by luminescence and electrochemical beads immunoassays, Mann-Whitney U test was performed, and statistically significant levels were considered at p < 0.05. The diagnostic capacity of each individual protein as well as the combination of markers was evaluated by a receiver operating characteristic (ROC) curve. ROC curves, their corresponding area under the curve (AUC) and maximized sensitivity and specificity values were calculated using the R package Epi 58.

## Supplementary Material

Supplementary figure and tables.

## Figures and Tables

**Table 1:** Samples used in the study.

Classification		Age (years) ± SD	Sample size (Male/Female)
**Control**	Negative colonoscopy/asymptomatic	44±7	11/19
	Other cancers	58±12	16/24
**Pathological**	Premalignant	60±7	16/9
	CRC	70±11	13/13
