# Supplementary material for: Multiplexed monitoring of a novel autoantibody diagnostic signature of colorectal cancer using HaloTag technology-based electrochemical immunosensing platform
Source: Theranostics. 2020 Feb 10;10(7):3022–34. doi: 10.7150/thno.42507 (PMC7053203; doi:10.7150/thno.42507)
Supplement: Supplementary file 1 — Supplementary figure and tables. [file thnov10p3022s1.pdf]

Multiplexed monitoring of a novel autoantibody diagnostic signature of colorectal cancer using HaloTag technology-based electrochemical immunosensing platform

María Garranzo-Asensio[1,2], Ana Guzmán-Aránguez[1], Eloy Povedano[3], Víctor Ruiz-Valdepeñas Montiel[3], Carmen Poves[4], María Jesús Fernandez-Aceñero[5], Ana Montero-Calle[2], Guillermo Solís-Fernández[2], Servando Fernandez-Diez[4], Jordi Camps[6], Meritxell Arenas[6], Elisabeth Rodríguez-Tomàs[6,7], Jorge Joven[6], Maricruz Sanchez-Martinez[2], Nuria Rodriguez[8], Gemma Dominguez[9], Paloma Yáñez-Sedeño[3], José Manuel Pingarrón[3,*,] Susana Campuzano[3,*], Rodrigo Barderas[2,*].

***** Co-senior authors and co-corresponding authors.

1. Departamento de Bioquímica y Biología Molecular, Facultad de Ciencias Químicas, Universidad Complutense de Madrid, 28040 Madrid, Spain.
2. UFIEC, Chronic Disease Programme, Instituto de Salud Carlos III, Majadahonda 28222, Madrid, Spain.
3. Departamento de Química Analítica, Facultad de Ciencias Químicas, Universidad Complutense de Madrid, 28040 Madrid, Spain
4. Gastroenterology Unit, Hospital Universitario Clínico San Carlos, E-28040, Madrid, Spain
5. Pathology Department, University Hospital Gregorio Marañon, E-28007 Madrid, Spain
6. Unitat de Recerca Biomèdica, Hospital Universitari Sant Joan, Institut d´Investigació Sanitària Pere Virgili, Universitat Rovira i Virgili, Reus (Spain).
7. Department of Radiation Oncology, Hospital Universitari Sant Joan, Institut d´Investigació Sanitària Pere Virgili, Universitat Rovira i Virgili, Reus (Spain).
8. Medical Oncology Department, Hospital Universitario La Paz, E-28046, Madrid, Spain.
9. Departamento de Medicina, Facultad de Medicina, Instituto de Investigaciones Biomédicas "Alberto Sols", CSIC-UAM, E-28029, Madrid, Spain.

*****To whom correspondence should be addressed:

Rodrigo Barderas.

Functional Proteomics Unit, UFIEC, Chronic Disease Programme, Instituto de Salud Carlos III, E-28222 Majadahonda, Madrid, Spain; Tel.: 34-91-8223231; E-mail: r.barderasm@isciii.es

Susana Campuzano

Departamento de Química Analítica, Facultad de Ciencias Químicas, Universidad Complutense de Madrid, 28040 Madrid, Spain; Tel.: 34-91-3944219; E-mail: susanacr@ucm.es

José M. Pingarrón

Departamento de Química Analítica, Facultad de Ciencias Químicas, Universidad Complutense de Madrid, 28040 Madrid, Spain; Tel.: 34-91-3944315; E-mail: pingarro@quim.ucm.es

Supplementary Tables and figures

Table S1. Individual clinical information of the colorectal cancer patients and controls used in the study.

Plasma code	Clasification	Group	Gender	Age	Cancer stage
CT1	Negative colonoscopy	Asymptomatic/Control	F	43	-
CT2	Negative colonoscopy	Asymptomatic/Control	M	35	-
CT3	Negative colonoscopy	Asymptomatic/Control	F	48	-
CT4	Negative colonoscopy	Asymptomatic/Control	M	41	-
CT5	Negative colonoscopy	Asymptomatic/Control	M	36	-
CT6	Negative colonoscopy	Asymptomatic/Control	F	46	-
CT7	Negative colonoscopy	Asymptomatic/Control	F	40	-
CT8	Negative colonoscopy	Asymptomatic/Control	F	41	-
CT9	Asymptomatic	Asymptomatic/Control	M	41	-
CT10	Asymptomatic	Asymptomatic/Control	F	55	-
CT11	Asymptomatic	Asymptomatic/Control	M	49	-
CT12	Asymptomatic	Asymptomatic/Control	F	44	-
CT13	Asymptomatic	Asymptomatic/Control	F	52	-
CT14	Asymptomatic	Asymptomatic/Control	M	31	-
CT15	Asymptomatic	Asymptomatic/Control	F	38	-
CT16	Asymptomatic	Asymptomatic/Control	F	41	-
CT17	Asymptomatic	Asymptomatic/Control	F	46	-
CT18	Asymptomatic	Asymptomatic/Control	F	37	-
CT19	Negative colonoscopy	Asymptomatic/Control	M	57	-
CT20	Negative colonoscopy	Asymptomatic/Control	M	58	-
CT21	Asymptomatic	Asymptomatic/Control	F	38	-
CT22	Asymptomatic	Asymptomatic/Control	F	51	-
CT23	Asymptomatic	Asymptomatic/Control	F	29	-
CT24	Asymptomatic	Asymptomatic/Control	F	50	-
CT25	Asymptomatic	Asymptomatic/Control	M	39	-
CT26	Asymptomatic	Asymptomatic/Control	F	45	-
CT27	Asymptomatic	Asymptomatic/Control	F	47	-
CT28	Asymptomatic	Asymptomatic/Control	F	51	-
CT29	Asymptomatic	Asymptomatic/Control	M	34	-
CT30	Asymptomatic	Asymptomatic/Control	M	40	-
B1	Breast cancer	Control	F	61	III
B2	Breast cancer	Control	F	50	II
B3	Breast cancer	Control	F	55	I
B4	Breast cancer	Control	F	57	II
B5	Breast cancer	Control	F	72	II
B6	Breast cancer	Control	F	60	I
B7	Breast cancer	Control	F	50	II
B8	Breast cancer	Control	F	68	I

B9	Breast cancer	Control	F	34	II
B10	Breast cancer	Control	F	46	III
B11	Breast cancer	Control	F	69	II
B12	Breast cancer	Control	F	79	III
B13	Breast cancer	Control	F	55	I
B14	Breast cancer	Control	F	41	I
B15	Breast cancer	Control	F	83	II
B16	Breast cancer	Control	F	65	I
B17	Breast cancer	Control	F	65	II
B18	Breast cancer	Control	F	47	I
B19	Breast cancer	Control	F	54	III
B20	Breast cancer	Control	F	55	III
L1	Lung cancer	Control	M	84	I
L2	Lung cancer	Control	M	72	II
L3	Lung cancer	Control	M	55	III
L4	Lung cancer	Control	M	55	III
L5	Lung cancer	Control	M	59	III
L6	Lung cancer	Control	M	85	I
L7	Lung cancer	Control	M	68	II
L8	Lung cancer	Control	F	63	III
L9	Lung cancer	Control	M	67	III
L10	Lung cancer	Control	M	71	III
L11	Lung cancer	Control	M	75	I
L12	Lung cancer	Control	F	53	III
L13	Lung cancer	Control	M	84	I
L14	Lung cancer	Control	M	82	I
L15	Lung cancer	Control	M	65	II
L16	Lung cancer	Control	M	64	III
L17	Lung cancer	Control	F	57	II
L18	Lung cancer	Control	M	79	I
L19	Lung cancer	Control	F	59	II
L20	Lung cancer	Control	M	71	III
PRE1	Adenoma	Premalignant group/pathological	M	59	-
PRE2	Adenoma	Premalignant group/pathological	F	64	-
PRE3	Adenoma	Premalignant group/pathological	M	65	-
PRE4	Adenoma	Premalignant group/pathological	M	60	-
PRE5	Adenoma	Premalignant group/pathological	M	59	-
PRE6	Adenoma	Premalignant group/pathological	M	57	-
PRE7	Adenoma	Premalignant group/pathological	M	70	-
PRE8	Adenoma	Premalignant group/pathological	M	40	-
PRE9	Adenoma	Premalignant group/pathological	F	57	-
PRE10	Adenoma	Premalignant group/pathological	M	60	-
PRE11	Adenoma	Premalignant group/pathological	F	50	-
PRE12	Adenoma	Premalignant group/pathological	M	67	-
PRE13	Adenoma	Premalignant group/pathological	F	61	-
PRE14	Adenoma	Premalignant group/pathological	F	62	-

PRE15	Adenoma	Premalignant group/pathological	M	59	-
PRE16	Adenoma	Premalignant group/pathological	F	54	-
PRE17	Adenoma	Premalignant group/pathological	M	60	-
PRE18	Adenoma	Premalignant group/pathological	M	69	-
PRE19	Adenoma	Premalignant group/pathological	M	60	-
PRE20	Adenoma	Premalignant group/pathological	M	62	-
PRE21	Adenoma	Premalignant group/pathological	F	60	-
PRE22	Adenoma	Premalignant group/pathological	F	75	-
PRE23	Adenoma	Premalignant group/pathological	M	61	-
PRE24	Adenoma	Premalignant group/pathological	F	59	-
PRE25	Adenoma	Premalignant group/pathological	M	57	-
CRC1	CRC	CRC group/pathological	F	85	IIIC
CRC2	CRC	CRC group/pathological	F	74	IV
CRC3	CRC	CRC group/pathological	M	63	IIIA
CRC4	CRC	CRC group/pathological	F	60	IIIA
CRC5	CRC	CRC group/pathological	M	75	IIIC
CRC6	CRC	CRC group/pathological	F	74	IV
CRC7	CRC	CRC group/pathological	M	82	IV
CRC8	CRC	CRC group/pathological	M	85	III
CRC9	CRC	CRC group/pathological	F	58	IV
CRC10	CRC	CRC group/pathological	M	54	IV
CRC11	CRC	CRC group/pathological	F	87	IV
CRC12	CRC	CRC group/pathological	M	84	IV
CRC13	CRC	CRC group/pathological	F	58	IV
CRC14	CRC	CRC group/pathological	F	51	IV
CRC15	CRC	CRC group/pathological	M	84	IV
CRC16	CRC	CRC group/pathological	M	62	III
CRC17	CRC	CRC group/pathological	M	71	IIIA
CRC18	CRC	CRC group/pathological	M	72	IV
CRC19	CRC	CRC group/pathological	F	55	IV
CRC20	CRC	CRC group/pathological	M	78	IIIA
CRC21	CRC	CRC group/pathological	M	61	IIIB
CRC22	CRC	CRC group/pathological	F	66	IIIA
CRC23	CRC	CRC group/pathological	F	59	IV
CRC24	CRC	CRC group/pathological	M	82	IV
CRC25	CRC	CRC group/pathological	F	66	IV
CRC26	CRC	CRC group/pathological	F	66	IV

Table S2. Analysis of the diagnostic value of indicated autoantibodies by means of ROC curves analyses.

	All controls *vs* Pathological			All controls *vs* CRC			All controls *vs* Premalignant		
**Autoantibody target**	**AUC (%)**	**Sensitivity (%)**	**Specificity (%)**	**AUC (%)**	**Sensitivity (%)**	**Specificity (%)**	**AUC (%)**	**Sensitivity (%)**	**Specificity (%)**
GTF2B	60.6	26.0	100.0	69.1	44.0	92.9	52.1	16.0	100.0
MAPKAPK3	72.5	50.0	95.7	75.8	56.0	95.7	69.1	44.0	95.7
PIM1	81.3	68.0	92.9	87.2	76.0	97.1	75.4	56.0	94.3
PKN1	77.1	70.0	80.0	83.5	76.0	81.4	70.7	64.0	80.0
SRC	70.1	64.0	82.9	75.9	72.0	84.3	64.3	56.0	82.9
STK4	75.9	68.0	82.9	76.1	72.0	82.9	75.7	68.0	80.0
SULF1	72.2	64.0	81.4	77.7	64.0	85.7	66.7	60.0	81.4
p53	57.4	36.0	87.1	62.2	44.0	87.1	52.7	36.0	80.0
Combination of statistically significant proteins	87.6	72.0	92.9	91.8	76.0	98.6	83.0	84.0	75.7
	**Asymptomatic *vs* Pathological**			**Asymptomatic *vs* CRC**			**Asymptomatic *vs* Premalignant**		
**Autoantibody target**	**AUC (%)**	**Sensitivity (%)**	**Specificity (%)**	**AUC (%)**	**Sensitivity (%)**	**Specificity (%)**	**AUC (%)**	**Sensitivity (%)**	**Specificity (%)**
GTF2B	63.8	26.0	100.0	71.4	56.0	83.3	70.3	53.8	83.3
MAPKAPK3	71.9	58.0	90.0	75.4	64.0	90.0	76.0	65.4	90.0
PIM1	78.9	62.0	93.3	85.1	76.0	93.3	85.5	76.9	93.3
PKN1	71.4	62.0	76.7	77.6	68.0	76.7	77.6	69.2	76.7
SRC	62.1	62.0	73.3	68.1	72.0	73.3	68.8	73.1	73.3
STK4	73.4	68.0	80.0	73.8	72.0	80.0	74.3	73.1	80.0
SULF1	64.8	72.0	53.3	69.3	88.0	46.7	70.4	88.5	46.7
p53	63.1	36.0	90.0	67.3	69.0	76.6	68.2	61.5	76.7
Combination of statistically significant proteins	85.3	74.0	90.0	92.4	76.0	96.7	78.4	72.0	80.0

Table S3. Comparison of the AUC obtained in different studies.

	Here described methodology			Previous methodologies		
	**Asymptomatic healthy control individuals vs CRC**					
**Autoantibody target**	**AUC (%)**	**Sensitivity (%)**	**Specificity (%)**	**AUC (%)**	**Sensitivity (%)**	**Specificity (%)**
GTF2B	71.4	56.0	83.3	66.0*	69.0*	60.0*
GTF2B	71.4	56.0	83.3	62.9^	13.0^	90.0^
MAPKAPK3	75.4	64.0	90.0	64.4^	8.0^	90.0^
PIM1	85.1	76.0	93.3	63.6	13	90.0
PKN1	77.6	68.0	76.7	-	-	-
SRC	68.1	72.0	73.3	-	-	-
STK4	73.8	72.0	80.0	64.4^	18.0^	90.0^
SULF1	69.3	88.0	46.7	-	-	-
p53	67.3	69.0	76.6	62.0*	71.0*	40.0*
p53	67.3	69.0	76.6	68.8^	13	90.0^

* Data from Barderas, R.; Villar-Vazquez, R.; Fernandez-Acenero, M. J.; Babel, I.; Pelaez-Garcia, A.; Torres, S.; Casal, J. I. Sci Rep 2013, 3, 2938.

^ Data from Villar-Vazquez, R.; Padilla, G.; Fernandez-Acenero, M. J.; Suarez, A.; Fuente, E.; Pastor, C.; Calero, M.; Barderas, R.; Casal, J. I. Proteomics 2016, 16, 1280-1290.

Table S4. Primary and secondary antibodies used in the study.

Primary Antibodies						
**Antibody target**	**Clonality**	**Source**	**Application**	**Dilution**	**Provider**	**Reference**
HaloTag	Monoclonal	Mouse	WB/ELISA	1:1000	Promega	G921A
p53	Monoclonal	Mouse	ELISA	1:1000	SCBT	sc-126
SRC	Polyclonal	Goat	ELISA	1:1000	R&D Systems	AF3389
MAPKAPK3	Monoclonal	Mouse	ELISA	1:1000	Abnova	H00007867-M02
**HRP-labeled secondary antibodies or HRP-labeled reagents**						
**Name**	**Clonality**	**Source**	**Application**	**Dilution**	**Provider**	
HRP anti-mouse IgG	Polyclonal	Goat	WB/ELISA	1:2500	Dako	
HRP anti-rabbit IgG	Polyclonal	Goat	WB	1:3000	BioRad	
HRP anti-goat IgG	Polyclonal	Rabbit	ELISA	1:1000	Jackson	
HRP anti-human IgG	Polyclonal	Goat	ELISA	1:10000	Jackson	

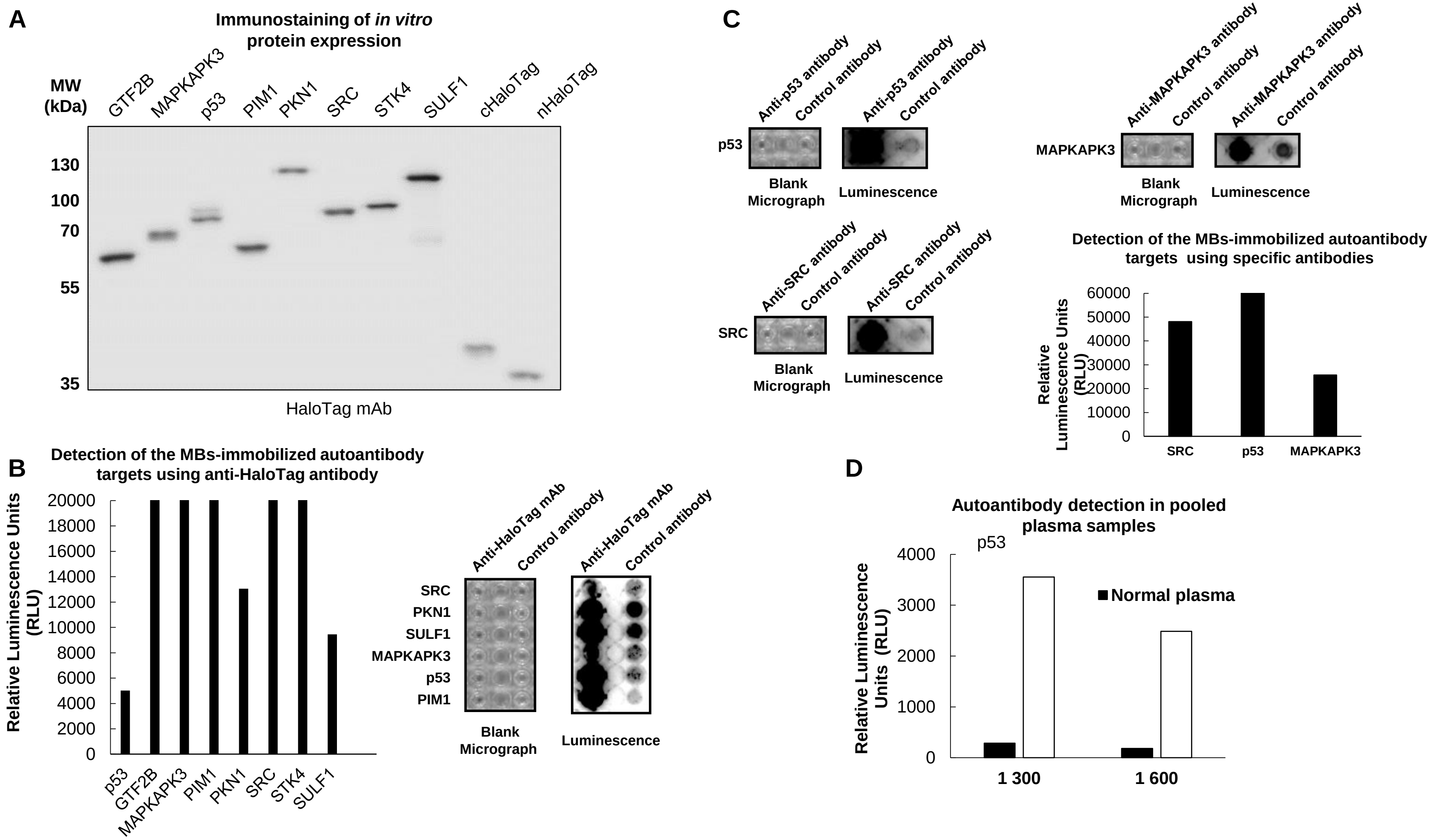

Figure S1. Verification of the *in vitro* protein expression and immobilization onto magnetic beads of the HaloTagged fusion proteins. (**A**) Autoantibody targets expression as HaloTag fusion proteins was verified by immunostaining using an antibody against the tag. The molecular mass of the proteins was in accordance to their theoretical mass with the HaloTag. (**B**) The correct immobilization of the proteins was verified by luminescence using an anti-HaloTag antibody. (**C**) The correct immobilization of the proteins was verified by luminescence using the indicated specific antibodies against the TAAs. (**B, C**) Quantitative luminescence was measured on The Spark multimode microplate reader (Tecan) and depicted by bar graph. Indicated MBs were placed on 96-well Maxisorp white plates and Luminescence was developed with ECL Western Blotting Substrate. Blank micrograph depicting the position of the indicated MBs, and luminescence recorded on an Amersham Imager 680 (GE Healthcare) are depicted. (**D**) Optimal assay conditions for the luminescence beads assay were found using pooled plasma samples at a 1:300 dilution.
